# Surgery

**Published:** 1896-03

**Authors:** W. M. Barclay


### SURGERY.

The **surgical treatment of Tic douloureux** in its severer and more inveterate forms has made extraordinary strides in the last few years, and the order of procedure in such treatment is liable to start up as a serious practical problem at any moment. The present seems, therefore, a fitting time to review our position.

The true pathology of the disease should naturally precede such an inquiry. Unfortunately this is by no means established. Dr. Gowers seems to "regard idiopathic neuralgia as the result of the over-action, the 'discharge,' of the nerve-cells constituting the proximate centre of the nerve;"[1] but admits that many of the phenomena suggest that a neuralgia which is at first purely central may, by the secondary vascular disturbance it induces, involve the nerve-sheaths. A large number of observers, however, have found well-marked histological changes in affected nerves, generally speaking of a chronic inflammatory type. Thus Horsley, Putnam, and Schweinitz[2] report swelling, shrinking, or disappearance of the axis-cylinders, a swollen condition of the medullary substance, proliferation of nuclei in the sheath of Schwann, and an increase of the endoneurium (sometimes also of the perineurium) in portions of excised nerves; and Mr. Rose in one of his cases[3] found an increase of the interstitial fibrous tissue between the ganglion-cells of the Gasserian ganglion he had removed. Keen[4] reports in the same sense. Dana—quoted by Rose[5]—thinks the majority of cases are due to "an obliterative endarteritis of the vessels supplying the nerve." Mr. Victor Horsley, writing of the surgical treatment of trigeminal neuralgia,[6] says: "I hold very strongly the opinion that epileptiform neuralgia is a purely peripheral malady, affecting principally the small subcutaneous branches of the nerve, or possibly the nerve endings, as well as the trunks of the fifth nerve, as they run in the bony canals of the facial bones, and that complete removal of the pain in any given division of the nerve may be obtained by ablation of the nerve from the base of the skull, unless the stump of the nerve become the seat of neuritis."

The various surgical measures that have been suggested and adopted are, in their historical order: (1) division of the nerve—neurotomy; (2) excision of a portion of the nerve trunk—neurectomy; (3) stretching the nerve; (4) ligature of the common carotid; (5) avulsion of the nerve—that is to say, tearing it off as close to its proximal end as possible, and cutting it off distally. **Neurotomy** and **nerve-stretching** need scarcely be discussed; for **excision** or **avulsion** of an affected nerve manifestly offers greater

[1] *Diseases of the Nervous System*, 2nd ed., vol. ii., 1893, p. 803.
[2] Quoted by Rose, *Brit. M. J.*, 1892, i. 53. [3] *Lancet*, 1890, ii. 914.
[4] *Am. J. M. Sc.*, 1896, cxi. 59.
[5] *Brit. M. J.*, 1892, i. 53. [6] *Brit. M. J.*, 1891, ii. 1251.

chances of a permanent result, and without any increased risk. **Ligature of the carotid** seems to be dismissed as an anachronism; although Mr. Rose[1] quotes some remarkable instances of its apparent success, and points out its interest on Dana's hypothesis of endarteritis. The risks, however, which attend this operation—especially of cerebral complications—are sufficient (in the uncertainty of its action) to negative its adoption, except perhaps as a last resort in special cases.

To these operative resources another has been added within the last few years; viz., more or less complete **removal of the Gasserian ganglion.** This bold measure has been conceived, and carried out in different ways, by several surgeons, and their suggestions and methods seem to be in a fair way of getting an influential and increasing following, and must therefore be reckoned with.

The real enquiry is narrowed to that of **the relative values,** or the proper spheres of action, **of neurectomy** (with which may be coupled avulsion) **and operations on the Gasserian ganglion.** Unfortunately a large number of cases in which neurectomy has been performed have relapsed after a longer or shorter interval, the pain either returning in the same branch or appearing in some other one. The first kind of relapse has been generally attributed to reunion of the nerve, from the removal of too small a piece; and no doubt in many cases this has been the cause. The regenerative power of nerves seems to be very considerable; and experiments of Hüter are quoted by Horsley[2] as showing that "not less than five inches must be removed" to prevent reunion with certainty, although he thinks this estimate excessive. On the other hand, he insists on factors of even greater importance in the causation of either kind of recurrence. The three branches of the trigeminal nerve lie for a considerable part of their course in small bony canals, and if the neuritis does not actually start in them, it is often very intense there; and, to ensure success, the section of the nerve should be made well to the proximal side of its entrance into such a canal. Further, in cases of long-standing neuralgia, the only chance of success by neurectomy would appear to be in getting as close as possible to the origin of the nerve, where it may still be healthy. This is the essential feature of the method of avulsion introduced by Thiersch, as long a piece of the nerve being torn away proximally as possible. In this way considerable lengths of nerve have been removed, and some remarkable success has been attained. Out of twenty-eight recorded operations, there had been only one relapse, and this in a very hysterical woman; and several of these had been performed six years previously.[3] Another cause of relapse, Mr. Horsley is convinced, is to be found in the occurrence of neuritis in the stump, generally due to failure of antiseptic precautions. In his paper, already alluded to, he gives a table of neurectomies performed by him on branches of

[1] *Loc. cit.* [2] *Loc. cit.* [3] Quoted by Rose, *loc. cit.*

the fifth nerve. Of ten cases, in which the last operation dates back three or more years, seven had remained quite free from pain for periods of from three to five years, one having been operated upon four times, the others only once. One of the three remaining cases was completely relieved when last heard of; the second (whose brother and sister were "melancholic") developed dementia after her third operation; and the last died of shock after avulsion of the fifth nerve-root from the bulb. Nine other cases are included in the table of more recent date. One remained quite free from pain 2 years 10 months, and three 2 years 6 months, after one operation; and *all* had been relieved up to the date of the publication—one, however, having undergone three operations. These results certainly represent a measure of success far greater than the conclusions of others would have led us to expect, and would seem to justify Mr. Horsley's conclusion, that operative procedure in these cases "is an imperative duty when all medical measures of relief have failed"; and that, if the principles laid down are followed, recurrence will occur far less often than it has hitherto.[1]

Mr. Rose,[2] on the other hand, after his elaborate review of the subject, gave it as his opinion "that in severe cases of epileptiform neuralgia both medical and surgical treatment had hitherto been unavailing to give permanent relief." For such inveterate cases of neuralgia, be they more or fewer, the question of **the possibility of removing the Gasserian ganglion** has been investigated by Rose, Hartley, Krause, and Andrews, apparently independently of one another. The first question was whether the ganglion could be removed entire. According to Hartley,[3] the ganglion lies between two layers of dura mater in a space—the cavum Meckelii—which extends from the trigeminal impression on the petrous bone "to the angle of the supra-orbital foramen, the foramen rotundum and ovale," passing external to the cavernous sinus. It reaches the posterior apex of the clinoid process behind, and laterally extends to the external border of the internal opening of the carotid canal. The superior portion is closely attached to the inner layer of the dura mater, while the inferior surface is only loosely connected with the outer layer of that membrane. "It is not so difficult to remove as one would imagine." Krause[4] and Keen[5] seem to have demonstrated its possibility in the living subject, and have published reproductions of photographs of the excised ganglion, with portions of the second and third divisions, the stump of the first, the sensory root, and (in Keen's case) what was taken for the motor root as well. Horsley,[6] on the contrary, in dissections undertaken to test its separability, found that, while he could raise the inferior division and the lower half of the ganglion from its bed without damage to the carotid artery

[1] *Loc. cit.* [2] *Brit. M. J.*, 1892, i. 264. [3] *Ann. Surg.*, 1893, xvii. 511.
[4] *Deutsche med. Wchnschr.*, 1893, xix. 341.
[5] *Am. J. M. Sc.*, 1896, cxi. 59. [6] *Loc. cit.*

in its canal or to the cavernous sinus, any attempt to strip off the upper half of the ganglion invariably resulted in a tear of the cavernous sinus. "I believe,," he writes, "that the operation of complete removal of the Gasserian ganglion is not possible, but that in the operation which Mr. Rose has subsequently described only a portion of it can be taken away."

If we assume that Krause and Keen have disproved this contention, there still remains a second question of no small importance: **Will complete extirpation of this ganglion lead to destructive changes in the eye?** It has been commonly thought that severance of the ophthalmic division of the fifth nerve from the Gasserian ganglion led to progressive disorganisation of the eyeball, while by some the ganglion seems to have been considered as a kind of "trophic" centre for its nutrition. Quite recently Turner[1] has made experiments in conjunction with Ferrier, with the object of discovering whether any "trophic" influence is exercised by the fifth nerve or its ganglion. The experiments included division of the fifth nerve-trunk (between the ganglion and the pons), its ophthalmic branch, and the intramedullary roots. Of the eighteen experiments, in all of which anæsthesia of the cornea was marked, only two showed signs of inflammatory destruction of the eyeball, and in both instances there was evidence of septic infection. In one case, five days after section of the right ophthalmic branch, both corneæ were touched with caustic; an ulcer formed in both, but in neither eye did it spread, and the process of healing was as satisfactory on the one side as on the other. In another case, eight days after section of the trunk behind the Gasserian ganglion, an irritant got into the anæsthetic eye; conjunctivitis and corneal opacity followed, but eventually disappeared with the exception of a small central ulcer, which persisted till death. Dr. Turner concludes from these experiments that "there is no evidence of trophic influence exerted by the Gasserian ganglion upon the cornea; and that, provided septic organisms are excluded, the ophthalmic branch may be safely divided or the Gasserian ganglion removed without fear of the disorganisation of the eye." It should be mentioned, however, that Gaule is quoted[2] as having arrived (from similar experiments) at the conclusion that trophic changes did result from, and were due to, lesion of the ophthalmic branch, or Gasserian ganglion, but that they did not follow lesion of the trunk behind the ganglion. Exception is taken to these results on the ground of the rapidity with which they followed the experimental lesion. If we turn to clinical records for guidance on this point, we seem to find ample corroboration of Dr. Turner's views. A glance at the table of operations on the Gasserian ganglion will show that in many cases no remark is made of any ill effects produced on the eye, while in many more it is explicitly stated that no "trophic" disturbance occurred. This evidence, however, is entirely

[1] *Brit. M. J.*, 1895, ii. 1279. [2] *Ibid.*

fallacious, so far at least as the Gasserian ganglion is concerned. In none of the thirty-seven cases (with two exceptions—12 and 26) was the whole Gasserian ganglion removed. Rose [1] makes no attempt to isolate the ophthalmic division, and pulls away the ganglion "piecemeal," getting away as much as he can with forceps and curette. Keen [2] says: "It is very doubtful to my mind whether by the method [Rose's] we do more than partially destroy the ganglion; certainly we do not 'remove' it. In only two of my cases were any ganglion cells found in the tissue removed from the supposed position of the ganglion, and yet I endeavored to break it up and remove it as completely as possible." Tiffany does "not attempt to take away all the ganglion." [3] The crucial test lies in those few cases in which the entire ganglion (as far as we are aware) has been taken away. The one case in which Keen accomplished this (12 in the table), and another (11) in which "the destruction of the ganglion was more complete than in the first four," stand out in strong contrast to his others, in which no trouble was induced in the anæsthetic eye, although no special precautions were taken to protect it. In the last two cases, and especially that of complete extirpation, the eye trouble was most serious and threatening, and in the latter instance relapsed on three successive occasions; and all this in spite of extra care and protection by occlusive dressing, and stitching of the eyelids together. These cases, if they stood alone, would point very strongly to some nutritive influence which was abrogated by an entire removal or destruction of the ganglion. Krause, however,[4] has reported eight cases of complete removal of the ganglion, with one death, and of the seven survivors none had any eye trouble except one patient, who was suffering from chronic conjunctivitis at the time of the operation. A corneal ulcer formed, but was cured. The question, therefore, must be left still undecided.

The **value of the operation** itself, whether complete or partial, and the question as to which of the two is the best method, must be left to the test of time. As some guide to the present position of the operation, I have drawn up the accompanying table of all the operations on the Gasserian ganglion of which I could get records. I have excluded all cases of intracranial neurectomy of the second and third divisions, which have been included in some accounts; a measure which deserves consideration on its own merits, and should not be confused with the more extensive procedure. I have also omitted Mr. Horsley's case of avulsion of the fifth nerve trunk from the bulb by trephining the skull, opening the dura mater, and lifting the brain off the tentorium. Only one of Krause's cases occurs in the table, because I could not find any record of the other seven. The mortality is high, but will probably be lessened, and in skilled hands is already much smaller. Of the thirty-seven cases

[1] *Loc. cit.* [2] *Loc. cit.* [3] *Ann. Surg.*, 1894, xix. 56.
[4] Quoted by Keen, *loc. cit.*

| Surgeon. | Reference. | Sex & Age. | Duration of Disease. | Previous Operative Treatment. |
|---|---|---|---|---|
| 1 Rose ... ... | *Brit. M. J.*, 1892, i. 263 | F. 60 | 7 years | (*a*) Aug. 19th, 1888. Dental nerve stretched through mouth. (*b*) March, 1889. Half-inch dental nerve excised by trephining lower jaw. (*c*) March, 1890. Dental and lingual divided close to base of skull. |
| 2 „ ... ... | „ „ | F. 63 | 10 years | All teeth, upper and lower, of right side extracted in 1884. |
| 3 „ ... ... | „ „ | F. 63 | 5 years | (*a*) Carious teeth removed. (*b*) Feb., 1890. Dental and gustatory divided close to base of skull. |
| 4 „ ... ... | „ „ | F. 37 | 12 years | (*a*) 1887. Half-inch dental nerve excised through trephine-opening in jaw. (*b*) July, 1890. Portions of dental and lingual removed close to base of skull. |
| 5 „ ... ... | „ „ | F. 37 | 7 years | Teeth extracted. |
| 6 „ ... ... | *Lancet*, 1892, ii. 953 | F. 56 | ... | ... ... ... ... |
| 7 Keen ... ... | *Am. J. M. Sc.*, 1896, cxi. 68 | M. 41 | 13 years | 13 operations, including removal of large part of upper jaw, as well as of various branches of nerves. |
| 8 „ ... ... | „ „ | M. — | 8 years | (*a*) All right upper teeth removed. (*b*) 1889. Subcutaneous neurectomy (twice). (*c*) March, 1890. Antrum drilled, and in April several polypi removed and septum of nose straightened. Neurectomy of infra-orbital. (*d*) May and Nov., 1890. Same nerve resected again. (*e*) Jan. 31st, 1891. Right carotid tied. |

| Operation on Gasserian Ganglion. | Effects on the Eye. | Other Notes. | Result. |
|---|---|---|---|
| 1 April 2nd, 1890. Ablation superior maxilla, trephining round foramen ovale and removal of ganglion. | Panophthalmitis—excision of eye-ball. | — | Complete relief, Jan. 28th, 1892. |
| 2 January 29th, 1891. Author's operation. | No trophic disturbance. | — | Complete relief, Feb., 1892. |
| October 29th, 1891. Author's operation. | For a time right eye painful and conjunctiva congested, but no ulcer. | February, 1892. Complained of inability to open mouth; remedied by forcible depression under anæsthetic. | Feb., 1892. Only complaint an occasional slight pain and intolerance of light in the right eye. |
| 4 November 5th, 1891. Author's operation. Two trephine openings made and joined into one; ganglion seen and even felt. | Two—three weeks after operation crop of small subepithelial vesicles on cornea, which burst leaving a superficial ulcer. This healed under suitable treatment. Eye-pad worn for six weeks. | A little cerebro-spinal fluid escaped when ganglion was removed. Some epistaxis and vomiting of altered blood, probably from wound of Eustachian tube. | No recurrence, Jan. 27th, 1892. |
| 5 January 16th, 1892. Author's operation. Dura mater wounded by trephine, exposing temporo-sphenoidal lobe. | — | Troublesome hemorrhage from pterygoid plexus; a small trephine opening. Thinks only the posterior half of the ganglion was removed. | Case only a few days old when reported. |
| 6 Author's operation. | — | — | Do. |
| 7 Hartley-Krause operation done in two stages; ganglion broken up. | No trouble. | Hemorrhage on lifting brain required packing with iodoform gauze (37 in. by 6 in.); left in three days. Respiration slowed & slight aphasia while it remained in. | Has had temporary twinges of pain, but otherwise quite relieved, twenty-six months after operation. |
| 8 June 18th, 1894. Same operation, also in two stages; ganglion broken up. | Eye had been destroyed before operation, and was enucleated on July 3rd. | Hemorrhage as in last case. | Nov., 1895. Writes that he had lately had a short attack of pain. |

| | Surgeon. | Reference. | Sex & Age. | Duration of Disease. | Previous Operative Treatment. |
|---|---|---|---|---|---|
| 9 | Keen ... ... | *Am. J. M. Sc.*, 1896, cxi. 68 | F. 63 | 12 years | (*a*) 1889. Neurectomy of dental nerve. (*b*) Feb., 1892. Dental and lingual nerves removed through vertical ramus. |
| 10 | " ... ... | " " | F. 60 | 5 years | Five operations; the last, division of second and third divisions at foramen rotundum and ovale. |
| 11 | " ... ... | " " | F. 54 | 12 years | (*a*) All teeth removed. (*b*) 1888. Half-inch dental nerve excised. (*c*) 1891. One-and-a-half inches dental avulsed, and removed superior maxillary nerve, dividing it well behind infra-orbital canal. |
| 12 | " ... ... | " " | M. 33 | 5 years | (*a*) March, 1895. Right upper teeth and alveolar process removed. (*b*) Since then three operations, including removal of infra-orbital nerve and opening of the antrum. |
| 13 | Lanphear ... | *Pacific M. J.*, 1892, xxxv. 647. Abstract in *Annual Univ. M. Sc.*, 1894, iii. A-69 | ... | ... | ... ... ... ... |
| 14 | Baker ... | *Am. J. M. Sc.*, 1894, cvii. 291 | M. 52 | 16 years | (*a*) Division of distal ends of the three divisions at their foramina of exit. |
| 15 | Caponotto... | *Policlin.*, Feb. 1, 1895. Abstract in *Brit. M. J.*, 1895, i., Epitome, p. 37 | M. 20 | From childhood | ... ... ... ... |

| Operation on Gasserian Ganglion. | Effects on the Eye. | Other Notes. | Result. |
|---|---|---|---|
| 9 February 20th, 1895. Same operation. | No trouble. | Assistant placed drill in his mouth, when Dr. Keen's back was turned. | Died in a week. Septic meningitis. |
| 10 May 23rd, 1895. Same operation, in two stages. Ganglion broken up. | No trouble. | The gauze plug measured 23 in. by 14 in. Removed on third day. Temperature was 100°—101° F. till fifth day. Slight discharge continued from the two ends of incision, and some pieces of bone discharged. | Entirely free from pain six months after. |
| 11 October 6th, 1895. Same operation. The second & third divisions tore away from ganglion, which was scooped out with curette. | Third day, eye red and corneal ulcer formed. It deepened and was very threatening; but with long-continued treatment and bandaging the eyes, she went home with "a distinctly healing ulcer." | Troublesome hemorrhage from posterior branch and trunk of middle meningeal. | Went home Nov. 27th, 1895. |
| 12 November 29th, 1895. Same operation; but as preliminary, eyelids stitched together. Ganglion removed entire with sensory and motor roots. | Stitches in eyelids taken out, on fourth day; cornea clear. Within 24 hours ulceration began. On sixth day lids re-stitched. In four days corneal irritation subsided, and the next day the lids separated. Eye protected by gauze-dressing. In two days after, ulceration again started. Lids stitched together again. "The eye is now improving." | On lifting brain, "furious hemorrhage" from middle meningeal—secured with difficulty. On tearing out ganglion profuse hemorrhage: supposed to be from aperture in cavernous sinus, whence 1st division was torn. Required packing with iodoform gauze. The temperature rose steadily, reaching 104° the next morning, and complained of bad headache. Both conditions relieved within an hour after removal of plug. | Quite free so far (three weeks). |
| 13 Rose's operation. | — | — | No recurrence, one year after. |
| 14 June, 1894. Rose's operation. | — | — | Died of shock in 38 hours. |
| 15 Ganglion reached by a combination of Rose's and Krönlein's operations. May, 1891. | — | A cholesteatoma, size of nut, found where root of trigeminus enters pons. Eustachian tube wounded. | Died on fourth day of acute septicæmia. Septic inflammation of wound, and meningitis. |

| | Surgeon. | Reference. | Sex & Age. | Duration of Disease. | Previous Operative Treatment. |
|---|---|---|---|---|---|
| 16 | Doyen ... | *Arch. prov. de Chir.*, iv. 7, 1895. Abstract in *Ann. Surg.*, 1896, xxiii. 73 | F. — | ... | ... ... ... ... |
| 17 | " ... ... | " " | ... | ... | ... ... ... ... |
| 18 | " ... ... | " " | ... | ... | ... ... ... ... |
| 19 | D'Antona ... | *Brit. M. J.*, 1893, i. 81 | F. — | ... | ... ... ... ... |
| 20 | Rogers ... | *Am.J.M.Sc.*, 1895, cx. 63 | M. 45 | 8 years | (*a*) Several right lower teeth extracted.<br>(*b*) All remaining teeth drawn.<br>(*c*) Nov., 1892. Neurectomy dental nerve in dental canal. |
| 21 | Park ... ... | *Med. News*, 1893, lxii. 183 | M. 53 | 5 years | ... ... ... ... |
| 22 | " ... ... | " " | F. 54 | ... | ... ... ... ... |
| 23 | Tiffany ... | *Ann. Surg.*, 1894, xix. 47 | F. 59 | 14 years | (*a*) Division of infra-orbital at its foramen. |
| 24 | " ... | " " | F. 79 | 30 years | No previous operations. |
| 25 | " ... | " " | F. 46 | 7 years | (*a*) 1882. Excision of one inch of dental nerve through maxilla. |
| 26 | Krause ... | *Deutsche med. Wchnschr.*, 1893, xix. 341. Abstract in *Brit.M.J.*, 1893, i., Epitome, p. 77 | F. 68 | 20 years | (*a*) Division of inferior dental nerve.<br>(*b*) Division of inferior maxillary trunk. |
| 27 | Parkhill ... | *Med. News*, 1893, lxiii. 319. Abstract in *Brit. M. J.*, 1893, ii., Epitome, p. 65 | F. 60 | 8 years | (*a*) Division of infra-orbital at its foramen.<br>(*b*) All three branches divided at their facial foramina.<br>(*c*) Another operation on second division |

| Operation on Gasserian Ganglion. | Effects on the Eye. | Other Notes. | Result. |
|---|---|---|---|
| 16 March 6th, 1893. Author's operation. | Function of eye intact. Vision perfect. | No trophic disturbance. | No recurrence two-and-a-half years after. |
| 17 Same operation. | — | | Died suddenly of apoplexy ten days after operation. |
| 18 Same operation. | — | — | Died in four days. No cause found except extreme feebleness of patient. |
| 19 December 19th, 1892. Modification of Rose's operation. | — | Epileptiform tic. Convulsive movements of hand and tongue—as many as 100 attacks a day. | Doing well. |
| 20 May 23rd, 1893. Rose's operation. Excision of the three divisions at ganglion, with partial destruction of latter. | It was two months before eye could be left uncovered without some irritation manifesting itself. | Ganglion could not be seen at operation. Aneurysm needle and sharp hook passed up to its presumed site, and "what seemed to be the ganglion" picked away. | No recurrence, Sept. 12th, 1893. |
| 21 Rose-Andrews operation, and avulsion of supra-orbital nerve through separate incision. | — | Tied common carotid for hemorrhage in pterygoid fossa. | ? |
| 22 Same procedures. | — | Five per cent. antipyrin spray used to check hemorrhage in maxillary fossa and skull. | ? |
| 23 September 8th, 1892. Hartley-Krause operation. The nerves and "as much of the ganglion as seemed to be attached to them" removed. | No disturbance in nutrition or function of eye. | Great flow of cerebro-spinal fluid, continuing some days; but wound healed in seven days. | No recurrence thirteen-and-a-half months after. |
| 24 June 30th, 1893. Hartley-Krause operation. | Do. | Flow of cerebro-spinal fluid. | No recurrence four months after. |
| 25 September 25th, 1893. Do. | Do. | Dura mater purposely opened to let off the cerebro-spinal fluid. This gave more room for manipulation. | Pain entirely relieved. |
| 26 January, 1893. Author's operation. The whole ganglion with nearly an inch of sensory root removed. | — | — | Quite relieved nine weeks after. |
| 27 September 14th, 1892. Rose's operation. | — | — | No recurrence ten months after |

| | Surgeon. | Reference. | Sex & Age. | Duration of Disease. | Previous Operative Treatment. |
|---|---|---|---|---|---|
| 28 | Finney ... | *Johns Hopkins Hosp. Bull.*, 1893, iv. 91. Abstract in *Brit. M. J.*, 1893, ii., Epitome, p. 101 | F. 47 | ... | ... ... ... ... |
| 29 | " ... | " " | M. 63 | ... | ... ... ... ... |
| 30 | " ... | " " | M. 69 | ... | ... ... ... ... |
| 31 | O'Hara ... | *Austral.M.J.*, 1893, xv. 513 | F. 66 | 5 years | All teeth extracted. |
| 32 | Novaro ... | *Riforma med.*, Nov. 7, 1893. Abstract in *Brit. M. J.*, 1893, ii., Epitome, p. 102 | ... | ... | ... ... ... ... |
| 33 | Gerster ... | *Med. Rec.*, 1895, xlvii. 803. Abstract in *Am. J. M. Sc.*, 1895, cx. 713 | M. — | ... | (*a*) Two years previously, Carnochan's operation on infra-orbital. (*b*) One year later, entire cicatrix removed for recurrence. |
| 34 | " ... | *Ann. Surg.*, 1896, xxiii. 58 | F. 34 | 7 years | (*a*) Nov., 1894. Excision of second branch (Lossen's operation). |
| 35 | Abbe ... ... | *Ann. Surg.*, 1896, xxiii. 60 | F. 65 | 10 years | ... ... ... ... |
| 36 | Andrews ... | *Internat. M. Mag.*, 1893, i. 479 | F. 62 | 5–6 years | ... ... ... ... |
| 37 | " ... | " " | F. — | 10 years | ... ... ... ... |

| Operation on Gasserian Ganglion. | Effects on the Eye. | Other Notes. | Result. |
|---|---|---|---|
| 28 Hartley-Krause operation. | — | — | Two months after complained of stiff and sore feeling on right side of head, but very different in character from the pain felt before. |
| 29 Do. | — | Severe and obstinate vomiting for 2–3 days after operation. | No pain one month after. |
| 30 Do. | — | — | Died in seven hours from heart-disease and shock. |
| 31 June 27th, 1893. Rose's operation. | — | Eyes washed with 1 in 25,000 corrosive sublimate solution, and eyelids sutured together. External meatus plugged. Slight cerebro-spinal escape at operation. Zygoma necrosed. | No return up to Sept. 15th, 1893. |
| 32 Beginning of 1891. Modification of Krönlein's operation — destroying ganglion at its inferior and posterior part. | — | During last year sensibility to pain, as well as to heat and touch, returned; but now the pain shoots from below upwards. | No recurrence. |
| 33 Hartley-Krause operation, in two stages. Ganglion removed in pieces. | — | Packing required for venous hemorrhage; removed, and operation completed on third day. Temperature rose to 102° F., and remained there. | Died on eighth day. After death no evidence of meningitis, but a focus of red softening in base of temporo-sphenoidal lobe. |
| 34 July, 1895. Hartley-Krause operation. Ganglion avulsed. | — | Much headache at first. A good quantity of serum escaped on removing gauze-drain on 5th day. Healed by August 23rd. | No recurrence up to Oct. 23rd, 1895. |
| 35 October 14th, 1895. Hartley-Krause operation. Ganglion curetted. | — | Packed wound for hemorrhage, removing gauze in 24 hours. Recovered well. | No recurrence of pain. Died in three months from another cause. |
| 36 Author's operation. | After a time ulceration of the cornea occurred, but healed. | Patient was requested to keep eye covered with a compress most of the time. | No return five months after. |
| 37 Do. | — | Not heard of since discharge, a month after operation. | Complete relief while under observation. |

seven died from the effects of the operation, or 18.9 per cent. The deaths were nearly equally distributed among three methods of operation—viz., Hartley-Krause method 3, Rose's method 2, and Doyen's method 2.[1] Dr. Keen [2] gives the mortality by Rose's method as 18 per cent., and by the Hartley-Krause method as only 9.8 per cent. My table shows no such superiority of one method over the other, and Rose himself has had no death. The next point of importance is the immunity from recurrence. This cannot yet be stated with any certainty, for time enough had not elapsed in any of the recorded cases to allow of a confident judgment. The longest interval of freedom is 2½ years, then 26 months (some temporary twinges—doubtful if true recurrence—No. 7), 22 months, a year or 13 months (three cases), and 10 months. But it is a significant fact that no single case has failed to be completely relieved after the operation, and in only 4 of the 37 cases had there been (up to the time of report) even the slightest relapse, and it was doubtful if any of the four exceptions were recurrences. Time, however, only can finally settle this point. Krause [3] concludes that this operation is justified only when (1) all the known sources of relief have been exhausted, but, above all, the lesser operation for neurectomy been tried; (2) the severity of the symptoms are such as (after such failure) to justify resort to an operation of this gravity. Nothing in the present state of our knowledge can be added to, or taken from, that statement.

The **ganglion can be exposed** by two methods: (1) that devised by Rose,[4] with which Professor Andrews's [5] is practically identical; and (2) the Hartley-Krause method, named after the two surgeons who independently planned an operation essentially the same. In the **Rose-Andrews operation** the foramen ovale is exposed in the pterygoid fossa, a disc of bone is removed by a trephine applied just outside the foramen, and the hole enlarged into the foramen and in any other direction required. The third division is used as a guide to the ganglion. The **Hartley-Krause method** [6] exposes the ganglion from above, the cranium being opened by an osteo-plastic flap in the temporal region. The dura mater is not opened, but is gently stripped from the middle fossa up to and over the ganglion and its divisions, the temporo-sphenoidal lobe being lifted up by a broad spatula. If it is wished to remove the ganglion entire, this should be seized first with a pair of artery forceps, and after it has been secured, the second and third divisions cut at their foramina, when, by a gentle but firm rotation of the forceps, the ganglion and its divisions can be avulsed, usually with the sensory root and the motor root as well. If the second and third divisions are cut before the ganglion is seized and avulsion made through

[1] Described in *Ann. Surg.*, 1896, xxiii. 69. It should be stated that these two deaths occurred in three operations. [2] Quoted from Krause, *loc. cit.*

[3] *Berl. klin. Wchnschr.*, 1892, xxix. 734. Quoted in *Annual Univ. M. Sc.*, 1893, iii. K-50. [4] *Loc. cit.* [5] *Internat. M. Mag.*, 1893, i. 479.

[6] Described by Keen, *loc. cit.*

them, they are very apt to tear off. In one of his cases Tiffany[1] thought he recognised the motor branch of the third division before cutting it, and suggests that an effort should be made to preserve it. This would prevent the paralysis of the muscles of mastication. It seems to have occurred not infrequently that in separating the ganglion the sub-arachnoid space has been opened with escape of the cerebro-spinal fluid, sometimes in large quantities. This has apparently not interfered with the healing of the wound, and Tiffany[2] even goes so far as to puncture the dura mater to let off the fluid, and finds he can get a better view with more room for the necessary manipulations. In the general treatment of these cases the chief point requiring notice is the protection of the eye. Rose and Keen both recommend closing the eye before beginning the operation, either by stitching the lids together with horse-hair, or by means of semi-lunar pieces of sticking-plaster fitted to each lid and tied together by silk tags.[3] Before closure of the lids the eye is to be washed with a 1 in 2000 solution of corrosive sublimate.[4] Both eyes should be kept covered for a week, but the eye on the side of the operation for three or four weeks at least. Mr. Rose further advises in his operation that the external meatus should be cleansed and plugged.

W. M. Barclay.

